# Deactivating Symmetry Breaking of a Soft Frank–Kasper Phase via Water-Induced Conformational Ordering of a Shapeshifting Dendritic Amphiphile

**DOI:** 10.1021/acsami.5c04140

**Published:** 2025-05-13

**Authors:** Chien-Lung Wang, Wei-Tsung Chuang, Mu-Tzu Lee, Yong-Rui Wang, Shih-Yong Chen, Hung-Ju Huang, Shao-Yuan Liu, Jhih-Min Lin, Chun-Yu Chen, Yao-Chang Lee, U-Ser Jeng

**Affiliations:** † Department of Chemistry, 202948 National Taiwan University, No. 1, Sec. 4, Roosevelt Rd, Taipei 10617, Taiwan; ‡ 57815 National Synchrotron Radiation Research Center, 101 Hsin-Ann Road, Hsinchu 30076, Taiwan; § Department of Applied Chemistry, 34914 National Yang Ming Chiao Tung University, 1001 Ta Hsueh Road, Hsinchu 30010, Taiwan

**Keywords:** soft frank-kasper phase, self-assembly, water, conformational space, folding-unfolding

## Abstract

Water guides biomolecules to their native conformations in a high-dimensional conformational space. To explore a similar role in synthetic self-assembly, a wedge-shaped, shape-shifting dendron (SD) was studied. Cooling from the isotropic melt trapped SD in a metastable Frank–Kasper σ phase with symmetry breaking due to high conformational freedom. In situ SAXS, microbeam SAXS, and ATR-FTIR confirmed that water vapor disrupts supramolecular micelles in the σ phase and unfolds some cone-shaped SD molecules, facilitating improved chain–chain packing and inducing the low-symmetry σ phase to transition into a more symmetric hydrated lamellar (*L*_w_) phase. The water-induced conformational ordering of the SD thus enables a rare σ → *L*_w_ phase transition, initially blocked by the SD’s hydrophilic volume fraction. Spectroscopy results also illustrated that during the conformational ordering, water molecules are encapsulated into the hydrophilic domains of the ordered phases, enhancing the phase stability of the *L*_w_ phase and the hydrated quasicrystalline DDQC and σ phases that evolved from the *L*_w_ phase. These findings reveal a novel self-assembly pathway for the wedge-shaped amphiphile that deactivates the symmetry breaking in the Frank–Kasper σ phase and emphasize the role of water in guiding synthetic molecules to their optimal supramolecular structures, echoing the self-assembly principles in nature.

## Graphical abstract

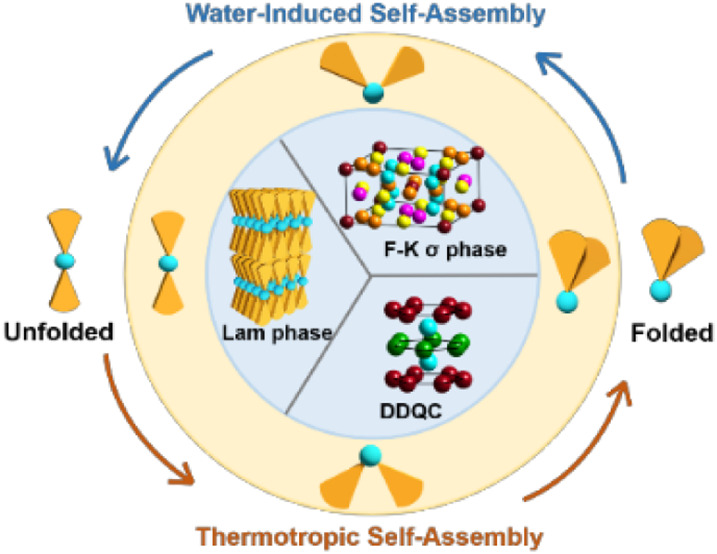

## Introduction

Water is known to play a crucial role in the self-assembly of biomolecules,^1,2^ as it guides the random walk of unfolded biomolecules through a complex conformational space toward unique energy-minimized folded structures.^3^ Although the energy landscape of the conformational space is multidimensional and contains many local energy minima that might trap a biomolecule in inactive conformations, studies have shown that the hydration of biomolecules helps smooth the energy landscape and allows the available thermal energy to push the biomolecules toward their native conformation.

Governed by interaction parameters and hydrophilic/hydrophobic volume ratios, synthetic amphiphiles self-assemble into basic supramolecular architectures such as spherical, cylindrical, lamellar, and interpenetrating network structures.^4–7^ Adding factors including complex molecular geometries,^8–11^ conformational asymmetry,^12–14^ size asymmetry,^15–18^ conformational freedom,^19–21^ etc., into the molecular design further allows the synthetic amphiphiles to demonstrate complex and dynamic self-assembly behaviors. Among the factors that increase supramolecular complexity, symmetry breaking has been recently recognized as an important one that enables the creation of aperiodic structures within crystallographic lattices.^22,23^ To uniformly distribute motifs in the crystalline domain under the restriction of lattice symmetry, in the soft Frank–Kasper (FK) phases, a fine balance between the chain-stretching penalty and interfacial tension is required.^22,23^ This competition results in distortions in the size and shape of the constituent supramolecular micelles in the unit cell and, consequently, symmetry breaking in the FK phases. However, it is often neglected that thermodynamically, the partially stretched chains could be metastable in the conformational space,^24^ and the complete chain-stretching of amphiphiles in the supramolecular micelles may ultimately result in structural ordering, which deactivates the symmetry breaking in the Frank–Kasper phase. Studies of lipids have shown that at the oil/water interface, the fully stretched aliphatic chains of lipids form liquid-ordered (*L*_o_) phases.^25–28^ This water-induced conformational ordering may allow chain-stretching to dominate in the competition with interfacial tension and consequently trigger unknown phase transitions of the soft FK phases, but the phenomenon has not yet been investigated in the literature.

To investigate whether water can guide synthetic amphiphiles toward the energy-minimized structure in the high-dimensional conformational space, as shown in Scheme 1, an amphiphilic shapeshifting dendron (SD) was synthesized. The SD is designed as a flexible wedge-shaped amphiphile because wedge-shaped amphiphiles are known to form various FK phases, in which the aliphatic chains of the amphiphiles are conformationally disordered and may be metastable. In addition to chain flexibility, high conformational freedom was also incorporated into the molecular architecture of the SD. As illustrated in Scheme 1, the planar angle (α) between the two hydrophobic dendrons of the SD can be switched between 0° and 180°, allowing the SD to alternate between a folded and an unfolded conformation.^29,30^ The unfolded conformation of the SD is particularly interesting because although wedge-shaped amphiphiles can easily shapeshift into fan-shaped or cone-shaped motifs to form either columnar or complex spherical phases,^29^ the self-assembly pathway for the direct phase transition between FK phases and lamellar (*L*) phases is inaccessible for most wedge-shaped amphiphiles. This is due to the fact that the formation of these two phases requires motifs with very distinct hydrophilic/hydrophobic volume ratios. Thus, the SD is purposely designed to expand the conformational space of the amphiphilic dendrons, enabling the investigation of whether water can manipulate the energy landscape along the self-assembly pathways of the SD. By combining thermal analysis, synchrotron X-ray, and spectroscopic characterization, it was revealed that water guides the SD from a metastable to a stable phase via conformational ordering. The water-induced conformational ordering activates an intriguing unfolding process of the SD and a rare phase transition between the FK σ phase and the *L* phase, as also illustrated in Scheme 1.

**Scheme 1:**
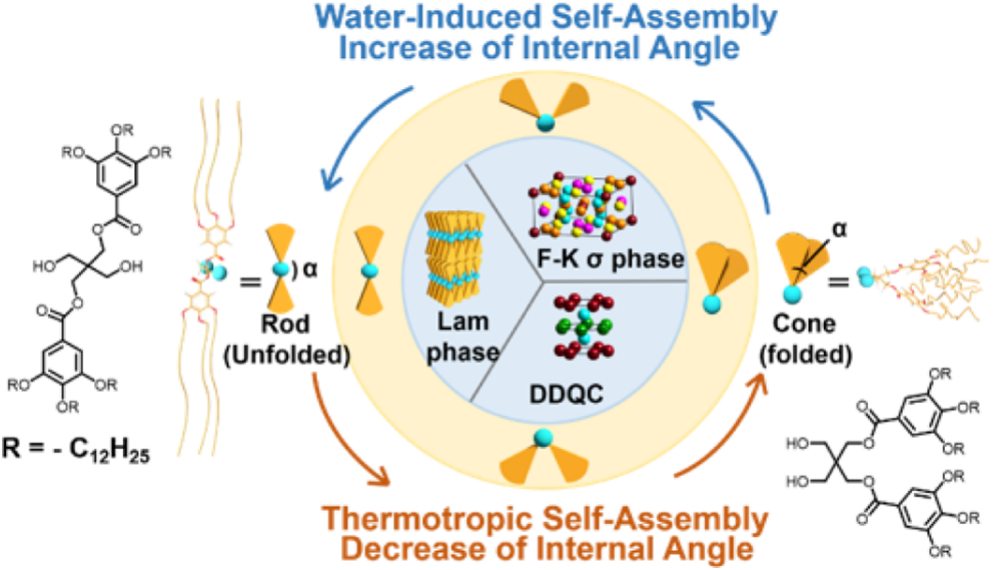
Illustrations of the Chemical Structure of the SD, Which Has Two Small Hydrophilic −OH Head Groups and Two Hydrophobic Dendrons, Its Rod-Like and Cone-Like Molecular Geometries and the Corresponding Self-Assembly Pathways^a^

a α is the planar angle between the two hydrophobic dendrons

## Results and Discussion

### Synthesis and Phase Behavior of the SD

Schemes S1–S2 show the synthetic route of the SD. The molecule serves as an intermediate of the Janus dendrimers.^31^ However, the shape-shifting self-assembly behavior of the SD was not observed previously because the conformational flexibility provided by the pentaerythritol core had been overlooked. The ^1^H NMR, ^13^C NMR, and mass spectra shown in Figures S1–S5 confirm the molecular structure of the SD. The phase behavior of the SD was first investigated in its dehydrated state. For the dehydrated SD, the differential scanning calorimetry (DSC) thermogram in Figure 1a shows that it underwent two endothermic phase transitions at 45.7 and 65.5 °C during the first heating scan, but only one at 66.5 °C during the second heating scan. The in situ temperature-dependent small-angle X-ray scattering (SAXS) data collected during the first heating process, shown in Figure 1b, indicate that at room temperature (RT), the SD exists in an *L* phase with a lamellar *d*-spacing of 49.1 Å. As the temperature increases, the SD undergoes sequential phase transitions to the dodecagonal quasicrystalline phase (DDQC), then FK σ phase, and eventually isotropizes at 66 °C. The characterization details and the indexes of the diffraction peaks of the DDQC phase and the σ phase can be found in Figure S6 and Table S1, eqs S1 and S2, Figure S7, Table S2, and eqs S3 – S4 and S7, respectively. The DSC thermogram in Figure 1a did not capture the DDQC-to-σ phase transition, likely due to the similar conformational disorder of the two phases.

**Figure 1:**
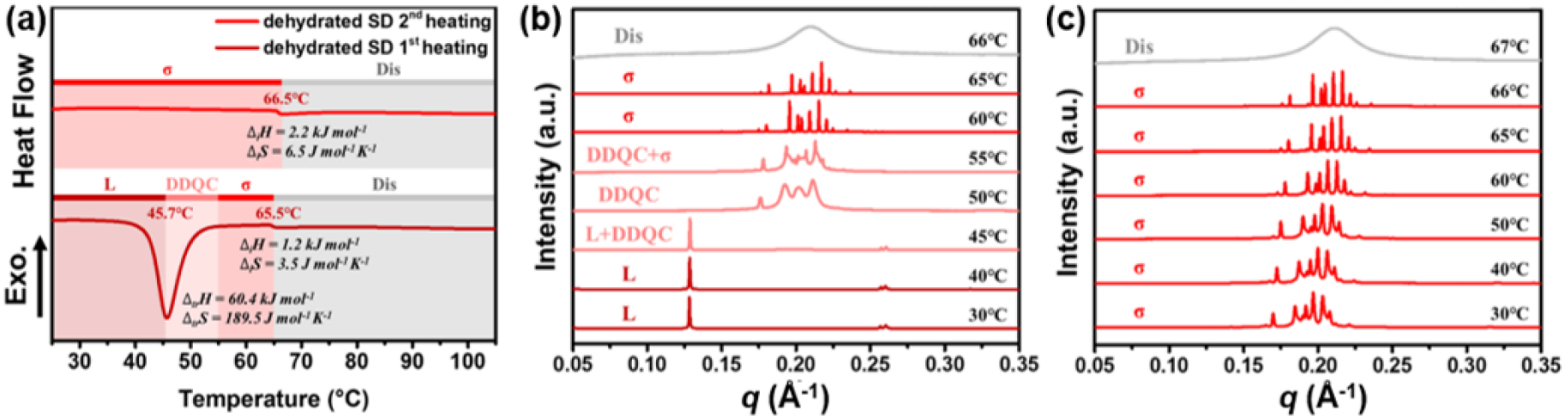
(a) DSC thermograms of the dehydrated SD. Temperature-dependent SAXS profiles of (b) the first heating scan and (c) the second heating scan of the dehydrated SD. Note: the scan rate in the DSC and in situ SAXS experiments is 10 °C min^–1^. The sample was annealed at each specific temperature for 5 min before the SAXS patterns were collected to ensure that the SAXS patterns are collected under thermodynamic equilibrium.

To our surprise, the cooling sequence did not return the SD back to its L phase. As seen in Figure 1c, the SD was still in FK σ phase after cooling the isotropic melt to RT. The second heating caused only the shrinkage of its lattice dimension (as the diffraction peaks shifted toward higher *q* values) but did not lead to any phase transition until the SD eventually isotropized at 67 °C. Although the σ phase seems to be stable at RT, the aging experiment in Figure 2a proves its metastability. The micrographs observed from the polarized light optical microscope (POM) show that exposing the σ phase to moisture caused the σ → *L* phase transition at RT. In the experiment, the SD was first heated to a temperature above *T*_i_ and then cooled to 25 °C to create the dehydrated σ phase. Under the POM, the σ phase shows very weak birefringence because SD molecules self-assemble into nearly spherical polyhedra, which give low birefringence. However, after 3 days under ambient conditions (25 °C, relative humidity (RH) = 75%), several birefringent spherulites of the *L* phase were observed in the original SD sample. The spherulites continued to grow until the σ phase completely transformed into the *L* phase after 5 days. The SAXS results in Figure S8 support the POM observation. It can be seen that as the exposure time increases, the diffraction signals from the L phase appeared at the expense of the disappearing of the σ phase. Moreover, the rate of the RT σ → *L* phase transition was found to be humidity-dependent. As seen in Figure 2a, when exposed to an environment with 100% RH, the σ phase took only 1 day to completely transform into the *L* phase. In contrast, it took about 30 days for the σ phase to slowly transform into the *L* phase if the sample was placed in a moisture-poor box with 30% RH. Thus, the σ → *L* phase transition of the SD is a water-induced phase transition since without water, the SD is deeply trapped in the conformational space with high metastability.

**Figure 2:**
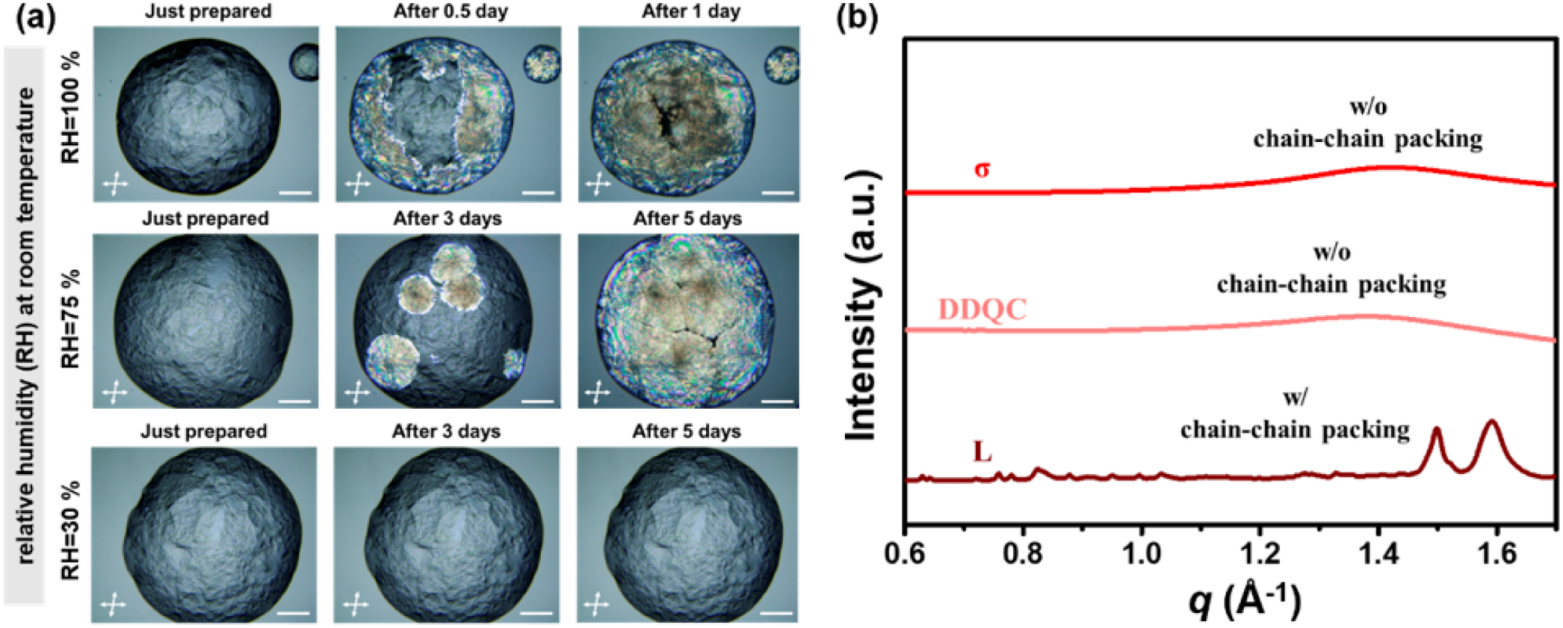
(a) POM micrographs of the RT σ → *L* phase transition of the SD under different RH. (b) The WAXS patterns of the *L*, DDQC, and σ phases. Note: (i) because the σ phase has low birefringence, to observe the σ phase and the lamellar phase (birefringent region) simultaneously, the polarizer and the analyzer of the POM were tilted slightly away from 90°. (ii) The scale bar represents 300 μm.

In the literature, phase transitions between the soft FK and columnar phases of wedge-shaped motifs are common, as the wedge-shaped motifs can easily switch between cone-shaped and fan-shaped conformations.^32,33^ Nevertheless, it is more difficult for the wedge-shaped motifs to form the *L* phase because their hydrophilic-to-hydrophobic volume ratio is far below 1:1.^34^ Thus, the water-induced σ → *L* phase transition was unexpected and requires an additional thermodynamic driving force to explain how the SD self-assembles into the *L* phase. To investigate the mechanism behind this transition, the wide-angle X-ray scattering (WAXS) patterns of the *L*, DDQC, and σ phases were collected, as shown in Figure 2b. The WAXS patterns indicate that the dodecyl chains are well-packed in the *L* phase but amorphous in the DDQC and σ phases. The results explain why a Δ_tr_ *H* of 60.4 kJ/mol was measured for the *L* → DDQC phase transition in the first DSC heating scan (Figure 1a), as the Δ_tr_ *H* represents the cost to randomize the conformations of the dodecyl units. However, during the cooling process, instead of restoring the conformational order of the *L* phase, the conformation-disordered σ phase was supercooled to RT. The long-range conformational order of the SDs was restored by the RT water-induced conformational ordering, as illustrated in Scheme S3a, which provides an enthalpic driving force to rearrange the disordered dodecyl units in the supramolecular micelles of the metastable σ phase into the well-packed structure of the *L* phase. By doing so, the chain–chain packing also reshapes the SD from the folded cone-shaped geometry to the unfolded rod-like geometry, facilitating the water-induced σ → *L* phase transition. The WAXS results also help to explain why the SD is trapped in the metastable σ phase, as stretching the dodecyl chains of the SD incurs an entropic penalty and poses a kinetic barrier for the σ → *L* phase transition. The RT metastable σ phase of the highly conformationally disordered SD is thus similar to biomolecules trapped in metastable structures within the conformation space, which are unable to overcome the energy barrier toward their native structures unless water smooths the energy landscape and allows the available thermal energy to drive the self-assembly toward the native conformations.

To better illustrate the phase behavior of the SD, an isobaric Gibbs free energy-temperature (G-T) plot is proposed in Scheme S3b. Based on the DSC and temperature-dependent SAXS/WAXS results, the SD demonstrates enantiotropic phase behavior, in which the *L* phase is the most stable phase below 45.7 °C. Upon heating, the sequential phase transitions of *L* → DDQC → σ → melt can be observed, as indicated by the orange arrows in the diagram. Nevertheless, the cooling process (shown by blue arrows) causes only the melt → σ phase transition at 65.5 °C, but it supercools the σ phase and does not bring the σ phase back to the *L* phase at ca. 45 °C. To enable the RT σ → *L* phase transition, water is necessary to activate the chain–chain packing of the dodecyl units and provide the enthalpic driving force for the SD molecules to escape from the metastable σ phase by tearing apart the polyhedra in the σ phase and rearranging the motifs into the *L* structure.

### Microscopic View of the Water-Induced σ → *L* Phase Transition

To understand the microscopic aspects of the RT σ → *L* phase transition, microbeam synchrotron 2D-SAXS was applied to first reveal the single-zone scattering pattern of the σ phase and then used to monitor the microstructures near the σ/L interface. The large-area single-crystalline domain of the σ phase was prepared by crystallizing the melt of the SD at *T* = 64 °C. As shown in Figure 3a, the domain size of the σ phase reached several hundreds of μm, enabling the collection of the single-zone microbeam 2D-SAXS pattern shown in Figure 3b. The Miller indexes of the diffraction spots can be identified based on the crystallographic information provided in Figure S7 and Table S2. The dehydrated σ phase of the SD belongs to the tetragonal crystal system, where *a* = *b* = 140.57 Å, *c* = 74.05 Å, and α = β = γ = 90°, as illustrated in Figure 3c. Each supramolecular micelle in the σ phase contains an average of 19 SD molecules, and the average spherical diameter of the micelles is ***d*** = 45.3 Å, calculated using the formulas eq.s S5 and S6. The zonal equation indicates that the microbeam 2D-SAXS pattern was obtained from the [110̅] zone of the σ phase (Figure 3d), where the (002) and (220) diffraction spots can be observed on axes that are perpendicular to each other in the reciprocal lattice revealed by the microbeam 2D-SAXS pattern.

**Figure 3:**
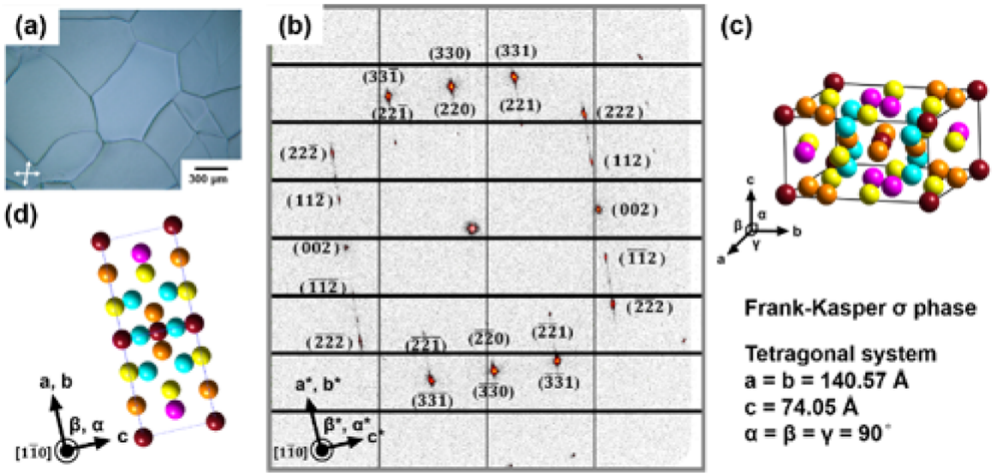
(a) The POM micrograph of the large-area single-crystalline grains of the σ phase obtained by crystallization at *T* = 64 °C. The micrograph was taken with the polarizer and analyzer tilted away from cross-polarization, because the crystalline grains show very weak birefringence. (b) Single crystal-like SAXS 2D pattern of the SD from the [110̅] zone. (c) The lattice parameters of the dehydrated σ phase of the SD. (d) Projection view of the σ phase along the [110̅] zone. Note: the diameter of the incident beam in the microbeam 2D-SAXS experiment is 10 μm.

Exposure of the dehydrated σ phase to ambient moisture resulted in the σ → *L* phase transition. Figure 4a,b shows the evidence in which birefringent spherulites of the *L* phase evolved from the σ phase. To reveal the relative orientation of the original σ phase and the later-evolved *L* phase, a series of microbeam 2D-SAXS patterns near the σ/*L* interface are collected and summarized in Figure 4c. It can be found that when the X-ray probing beam (beam size = 10 μm) locates at the σ/L interface, in addition to the diffraction signals of the σ phase, the (001) and (002) reflections of the L phase are also observed along the meridian of the 2D-SAXS patterns. Figure 5 shows one of the typical 2D-SAXS patterns at the σ/L interface, which reveals the relative orientation of the σ and L lattices. It can be seen that the σ and *L* phases have their *c*-axes perpendicular to each other. By orienting the real-space lattices of the σ and *L* phases according to the 2D pattern, it was found that the supramolecular micelles in the σ phase were cut open along the *c*-axis of the σ phase, and the disaggregated SD molecules were then rearranged to form the *L* phase, which has its lamellar normal perpendicular to the *c*-axis of the σ phase, as illustrated in the right panel of Figure 5.

**Figure 4:**
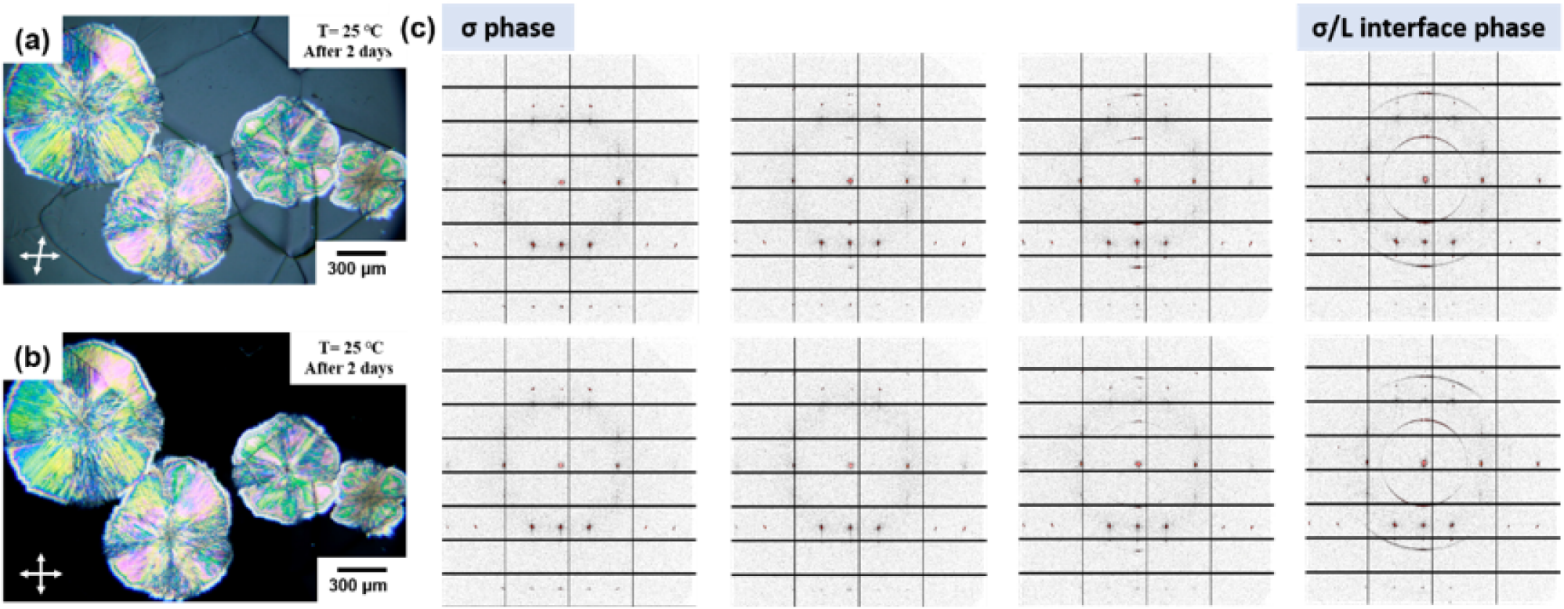
(a) OM and (b) POM micrographs of the σ phase sample that has been exposed to the ambient moisture for 2 days. The later-evolved spherulite of the *L* phase shows birefringence, but the original σ phase does not. (c) The microbeam SAXS patterns collected near the σ/*L* interface at the interval of 50 μm in the 2D mapping experiment to reveal the microstructures in the 200 μm × 150 μm area.

**Figure 5:**
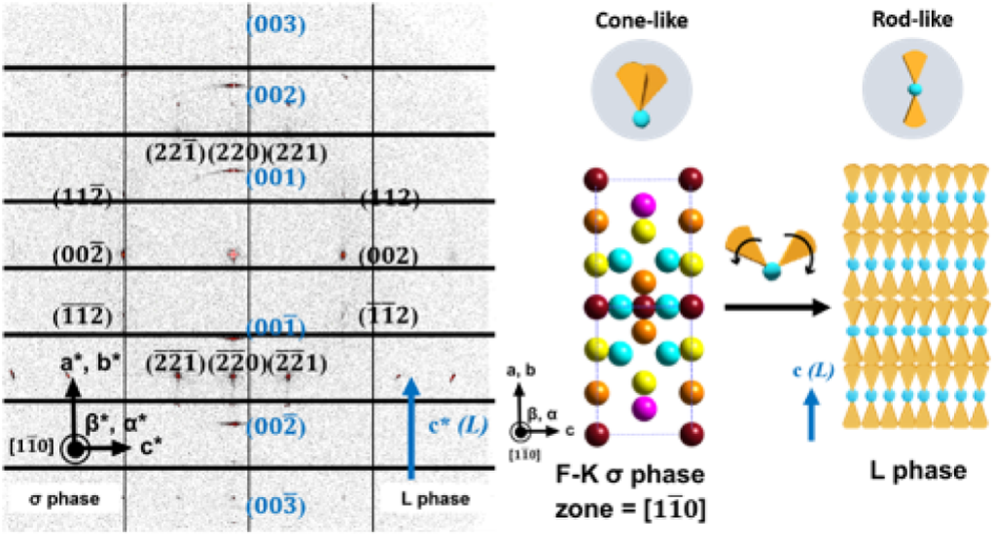
Typical microbeam SAXS pattern at the σ/L interface. The pattern shows that the lamellar normal is perpendicular to the *c*-axis of the σ phase, as illustrated by the packing models on the right panel.

### Influences of the Incorporated Water to the Hydrated Phases of the SD

To our surprise, the water-induced σ → *L* phase transition led the SD into a hydrated lamellar (*L*_w_) phase that is different from the original *L* phase. In Figure 6a, the *L*_w_ phase gives two endothermic peaks at 48 and 91.7 °C. The temperature-dependent SAXS patterns in Figure 6b indicate that the endothermic transition at 48 °C disordered the *L*_w_ phase and resulted in the amorphous halo at 0.188 Å^–1^ at 50 °C. Reorganization of the SD molecules occurs between 50 and 60 °C, leading to the diffraction signals of the hydrated DDQC (DDQC_w_) phase observed at 60 to 70 °C. Above 70 °C, the DDQC further transformed into the hydrated σ phase (σ_w_), and the σ_w_ phase eventually isotropizes at 91.7 °C. Compared to the phase behavior of the dehydrated *L* phase in Figure 1, the *L*_w_, DDQC_w_, and σ_w_ phases of the moisture-exposed sample have obviously elevated transition temperatures. ATR-FTIR spectra measured at beamline TLS14A1, National Synchrotron Radiation Research Center (NSRRC), Taiwan, show that the *L*_w_ and σ_w_ phases contain more water than the dehydrated *L* and σ phases, as indicated by the stronger O–H stretching band (3000–3700 cm^–1^) of the hydrated phases in Figure 6c. Therefore, according to the ATR-FTIR spectra, water was incorporated into the *L*_w_ phase during water-induced conformational ordering. Moreover, compared to the original dehydrated σ phase, the σ_w_ phase evolved from the *L*_w_ phase gives reflections at lower scattering angles (see Figure S9). The result confirms that water expands the lattice parameters of the σ_w_ phase to *a* = *b* = 142.10 Å and *c* = 75.84 Å. More importantly, this implies that the incorporated water molecules in the *L*_w_ phase were not evaporated during heating. Instead, water is preserved in the hydrophilic cores of the supramolecular micelles of the DDQC_w_ and σ_w_ phases during conformational disordering of the *L*_w_ phase. Our previous study shows that the embedded water molecules enhance the phase stability of the 1D hydrated artificial water channels by forming internal water-containing hydrogen-bonded networks.^35^ The elevated transition temperatures of the hydrated phases thus prove that the water molecules incorporated during the water-induced conformational ordering provide a similar stabilization mechanism to the 2D *L*_w_ and 0D DDQC_w_ and σ_w_ phases of the SD. The thermogravimetric analysis results in Figure S10a confirm that the σ_w_ phase loses about 3 wt % of the incorporated water at 60 °C for 10 min. Figure S10b shows the DSC thermograms of the σ, σ_w_, and σ_w_ phase that has been thermally annealed at 60 °C for 10 min. It was found that thermal annealing at 60 °C caused partial dehydration of the σ_w_ phase and decreased the isotropization temperature and Δ_tr_ *H* of the σ_w_ phase. These results further support the essential role of the incorporated water in the thermodynamic properties of the hydrated phases of the SD.

**Figure 6:**
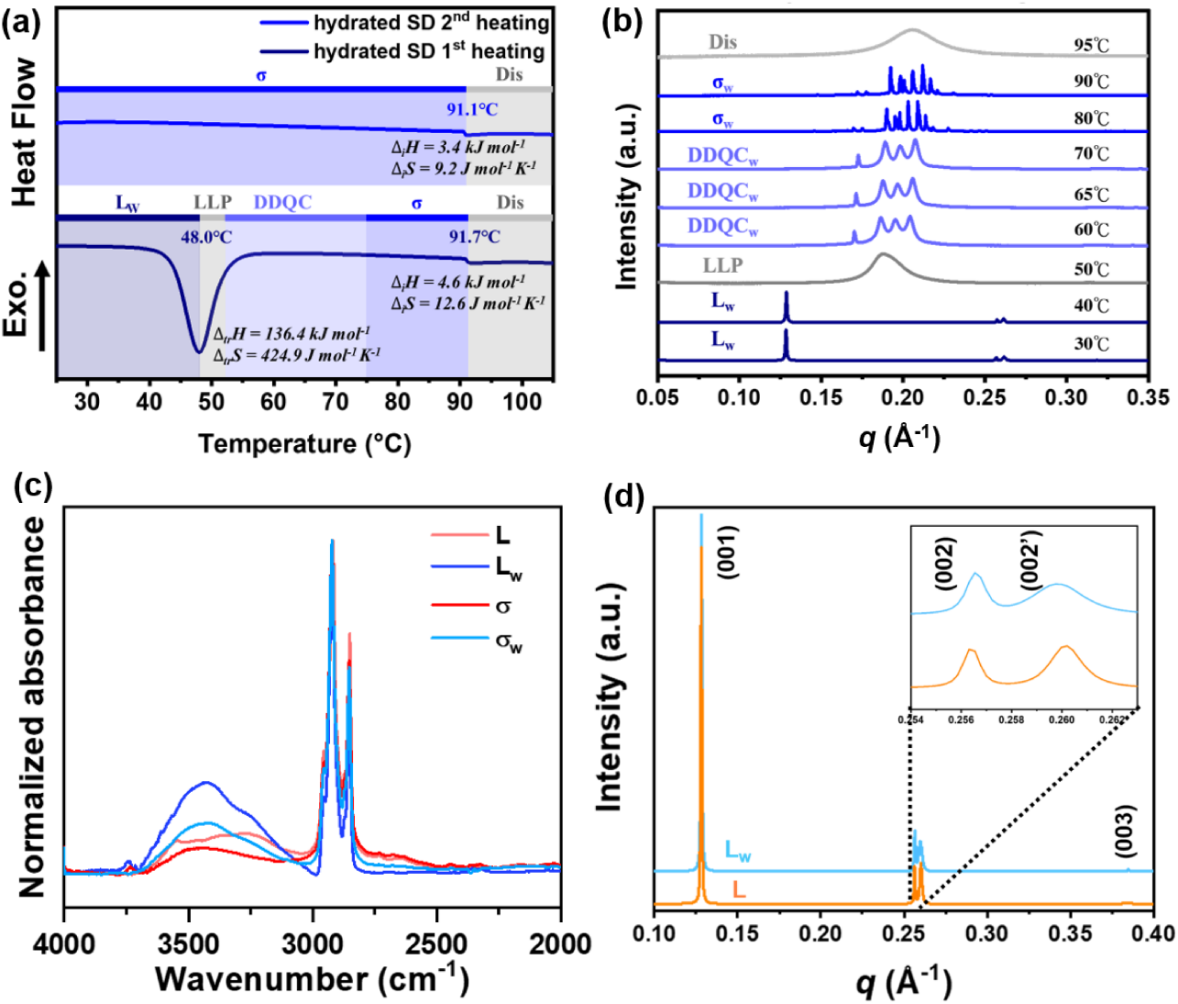
(a) DSC thermogram of the hydrated SD. (b) The temperature-dependent SAXS profiles of the first heating scan of the hydrated SD. (c) ATR-FTIR spectra of the SD in the dehydrated and hydrated *L* and σ phases. (d) 1D-SAXS profiles of the *L* and *L*_w_ phases of the SD. Note: the scan rate in the DSC and in situ SAXS experiments is 10 °C min^–1^. The sample was annealed at each specific temperature for 5 min before the SAXS patterns were collected to ensure that the SAXS patterns are collected under thermodynamic equilibrium. The *L* sample refers to the lamellar phase formed during solvent evaporation of the ethyl acetate solution of the SD. Note: the ATR-FTIR spectra were normalized using the CH_2_ stretching signals.

### The Folding-Unfolding Mechanism of the SD

The above characterization results, although confirming that the RT water-induced σ → *L*_w_ phase transition is thermodynamically feasible, do not change the fact that the hydrophilic-to-hydrophobic volume ratio of the SD is too small to form the *L*_w_ phase. Thus, how the wedge-shaped SD adjusts its molecular geometry to adapt to the supramolecular *L* and *L*_w_ structures still needs to be investigated. In fact, the *L* and *L*_w_ phases of SD are not typical lamellar phases. As can be seen in Figure 6d, the (002) reflection of the *L* and the *L*_w_ phases splits into two peaks: (002) and (002’), and in Figure S11, the WAXS patterns suggest that the aliphatic chains are packed more closely in the *L*_w_ phase. The *q* ratio of the (001) and (002) reflections is 1:2, indicating that the SD molecules self-assemble into a lamellar structure with a *d*-spacing of 49.1 Å. However, the (002’) reflection was observed at a larger scattering angle than (002), indicating another periodic structure with a smaller *d*-spacing than 49.1 Å is also present in the *L* and the *L*_w_ phases. To reveal this structure, the SAXS pattern of a sheared sample of the SD was measured and is shown as the gray trace in Figure 7a. It was found that the shearing process eliminated the (001) and (002) reflections but revealed the primary reflection of (002’). The (001’) and (002’) reflections give a *q* ratio of 1:2, showing that the additional periodic structure is also a lamellar structure with a slightly smaller *d*-spacing of 48.3 Å. The two lamellar structures are well-dispersed in the *L* and *L*_w_ phases since the POM micrographs did not detect macroscopic phase-separated domains. A possible mechanism to create two lamellar *d*-spacings in the *L* and *L*_w_ phases is proposed in Figure 7b. With high conformational flexibility, the SD molecules may adopt either the folded conformation to form bimolecular layers or the unfolded conformation to form unimolecular layers. The bimolecular layers of the folded SD create the larger *d* (folded) for the (001) and (002) reflections, whereas the unimolecular layers of the unfolded SD create the smaller *d* (unfolded) for the (001’) and (002’) reflections. In Figure 7a, the *L* and the *L*_w_ phases also exhibit different reflection intensities (*I*) from the (002) and (002’) reflections. The higher $I_{002^{’}}$ / $I_{002}$ ratio from the *L* phase indicates that when water is insufficient, more SD molecules are unfolded and adopt the rod-shaped conformation to support the layer structure in the *L* phase, whereas in the *L*_w_ phase, the SD molecules disaggregated from the supramolecular micelles of the σ phase prefer to remain folded, so that their hydrophilic −OH head groups are more exposed and interact better with water molecules. Thus, although the hydrophilic-to-hydrophobic volume ratio of the SD is unfavorable for the formation of the lamellar phases, the SD manages to utilize the folding-unfolding mechanism to create the optimal ratios of the wedge-shaped and rod-shaped conformers to stabilize the *L* and *L*_w_ phases.

**Figure 7:**
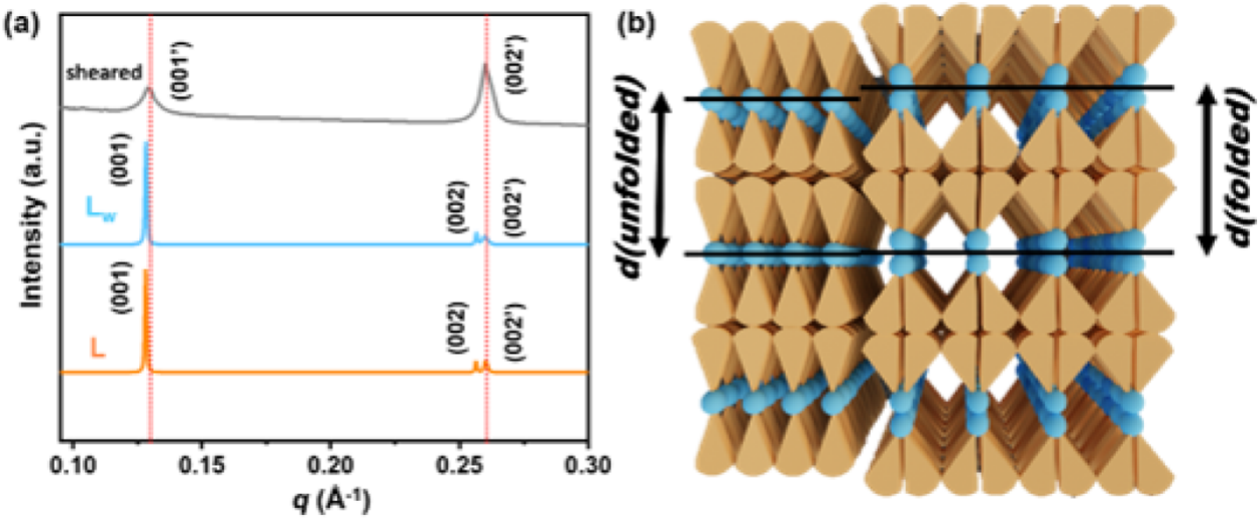
(a) 1D-SAXS profiles of the *L*, *L*_w_, and sheared lamellar phases of the SD. (b) Illustration of the coexistence of folded and unfolded SD in the *L* and *L*_w_ phases.

## Conclusion

In this study, a wedge-shaped shapeshifting dendron, SD, was synthesized to create a self-assembly system with a high-dimensional conformational space. Upon cooling, the high conformational freedom prevents the isotropic liquid of SD from reaching its thermodynamic equilibrium phase. Instead, the competition between the chain-stretching penalty and interfacial tension creates symmetry breaking and traps the SD in a metastable FK σ phase. Exposure of the σ phase to ambient moisture allows water to activate the conformational ordering and guide the SD toward the energy-minimized structure in the conformational space. In situ SAXS, microbeam SAXS, and ATR-FTIR spectra collected at the synchrotron facility confirm that the seemingly hydrophobic supramolecular micelles in the σ phase can be broken by the penetrated water vapor. The disrupted folded SD molecules then redeploy themselves, with some unfolded ones, into the more symmetrical lamellar planes along the [001] zone of the original σ phase in the RT water-induced σ → *L*_w_ phase transition. The water-induced phase transition not only deactivates the symmetry breaking in the FK σ phase but also encapsulates water molecules into the hydrophilic domains of the hydrated *L*_w_, DDQC_w_, and σ_w_ phases. DSC and in situ SAXS results confirm that the encapsulated water molecules significantly enhance the thermodynamic phase stability of these hydrated ordered phases. In the literature, the formation of the L phase is difficult for the wedge-shaped amphiphile due to the constraints from small hydrophilic volume fractions. The water-induced conformational ordering establishes a folding-unfolding mechanism that shapes a self-assembly pathway previously unseen in wedge-shaped amphiphiles. Similar to its role in the supramolecular chemistry of biomolecules, water smooths the energy landscape of the SD molecules and directs them toward their optimal structures, underscoring its essential role in both natural and artificial systems.

## Experimental Section

### Synthesis of SD

The SD was synthesized by connecting two hydrophobic arms (3,4,5-tris­(dodecyloxy)­benzoic acid (3,4,5-TDBA)) to a pentaerythritol core via Steglich esterification. The purity of the SD was checked by ^1^H NMR, ^13^C NMR, and HRFD-MASS (see Scheme S1, S2 and Figures S1–S5 for details).

### Differential Scanning Calorimetry (DSC)

The DSC analysis was carried out with a TA Instruments Q Series 20 Differential Scanning Calorimeter, paired with an RSC 90 cooling system to provide a low-temperature environment, and operated entirely under a nitrogen atmosphere. Approximately 3 mg of the sample was sealed in a Tzero Aluminum Pan with a lid, while an empty pan served as the reference. The process involved holding at 20 °C for 10 min, heating from 20 to 120 °C at 10 °C/min, holding at 120 °C for 10 min, cooling from 120 to −20 °C at 10 °C/min, and holding at −20 °C for 10 min. This cycle was repeated three times.

### Polarized Optical Microscopy (POM)

The Leica DM270 Polarized Optical Microscope, equipped with two polarizers, was used for the POM observation. The samples were prepared by placing them on a glass slide substrate, and the polarizers were then used to determine whether the samples exhibited birefringent properties.

### Small/Wide-Angle X-ray Scattering

SAXS and WAXS measurements were conducted at the Taiwan National Synchrotron Radiation Research Center (NSRRC) on the TPS BL13A beamline. The synchrotron light source operates at 5.6 to 25 keV, with an X-ray wavelength of 0.8265 Å and a beam focus size of 400 × 200 μm^2^. The scattering vector (*q*) range is 0.004 to 0.45 Å^–1^ for SAXS and 0.4 to 1.8 Å^–1^ for WAXS. Samples were prepared by taking approximately 1 mg of each sample and wrapping it in two layers of heat-resistant Kapton tape. For the in situ temperature-dependent experiments, the heating rate was set at 10 °C/min, with samples equilibrated for 5 min before measurement.

### Microbeam 2D Small-Angle X-ray Scattering

Microbeam 2D SAXS measurements were conducted at the NSRRC on the TPS BL 25A1 beamline. The synchrotron light source operates at 5.5 to 20 keV, with an X-ray wavelength of 1.000 Å and a beam focus size of 10 × 10 μm^2^. The scattering vector (*q*) range is 0.005 to 0.6 Å^–1^. Samples were prepared by placing approximately 1 mg of the sample on a single-layer glass slide. X-rays penetrated the sample, producing a two-dimensional scattering pattern, which was then processed by using SAXS software to obtain a one-dimensional scattering profile.

### Attenuated Total Reflection-Fourier Transform Infrared Spectroscopy (ATR-FTIR)

ATR-FTIR measurements were conducted at the NSRRC on the TLS BL 14A1 beamline. The IR light source is derived from an arc extracted from a bending magnet, with the apparatus covering a spectral range of 400 to 4000 cm^–1^ at a resolution of 0.125 cm^–1^, achieved over 128 scans.

## Supplementary Material



## Notes

The synthetic procedures of the SD, ^1^H and ^13^C NMR, and MASS spectrum of the SD, unit cell parameters, Miller indices, and SAXS patterns of the FK σ phase and DDQC phase of the SD (PDF)
