# Supplementary material for: Deactivating Symmetry Breaking of a Soft Frank–Kasper Phase via Water-Induced Conformational Ordering of a Shapeshifting Dendritic Amphiphile
Source: ACS Appl Mater Interfaces. 2025 May 13;17(21):31403–10. doi: 10.1021/acsami.5c04140 (PMC12123561; doi:10.1021/acsami.5c04140)
Supplement: Supplementary file 1 [file am5c04140_si_001.pdf]

Supporting Information

Deactivating Symmetry Breaking of a Soft Frank-Kasper phase via Water-Induced Conformational Ordering of a Shapeshifting Dendritic Amphiphile

Chien-Lung Wang[a]*, *Wei-Tsung Chuang*[b]*, *Mu-Tzu Lee*[c], *Yong-Rui Wang*[a], *Shih-Yong Chen*[a], *Hung-Ju Huang*[c], *Shao-Yuan Liu*[c], *Jhih-Min Lin*[b], *Chun-Yu Chen*[b], *Yao-Chang Lee*[b], *U-Ser Jeng*[b]

[a]*Department of Chemistry, National Taiwan University, No. 1, Sec. 4, Roosevelt Rd, Taipei 10617, Taiwan*

[b]*National Synchrotron Radiation Research Center, 101 Hsin-Ann Road, Hsinchu 30076, Taiwan*

[c]*Department of Applied Chemistry, National Yang Ming Chiao Tung University, 1001 Ta Hsueh Road, Hsinchu 30010, Taiwan*

All authors have given approval to the final version of the manuscript.

*** Corresponding Author**

Email: kclwang@ntu.edu.tw

*** Corresponding Author**

Email: weitsung@nsrrc.org.tw

1. Synthetic Procedures of the Shapeshifting Dendron (SD)

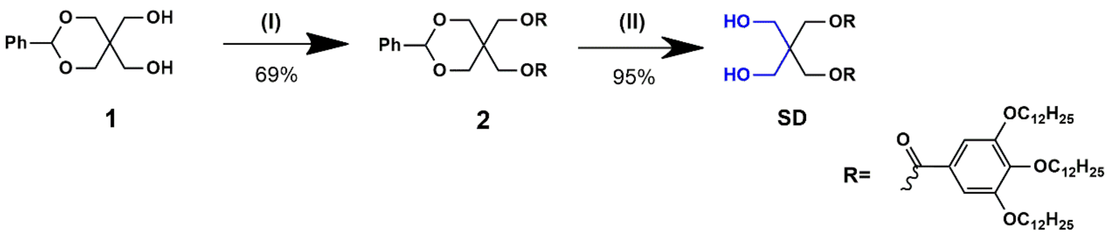

Scheme S1. The synthetic route of **SD**. (I) **3,4,5-Tris(dodecyloxy)benzoic acid(3,4,5-TDBA),** diisopropyl carbodiimide (DIPC), 4-(Dimethylamino)pyridinium 4-toluenesulfonate (DPTS), CH_2Cl_2, 25 °C, 16 hr; (II) Pd/C, H_2, CH_2Cl_2/MeOH = 2/1, 25 °C, 6 hr.

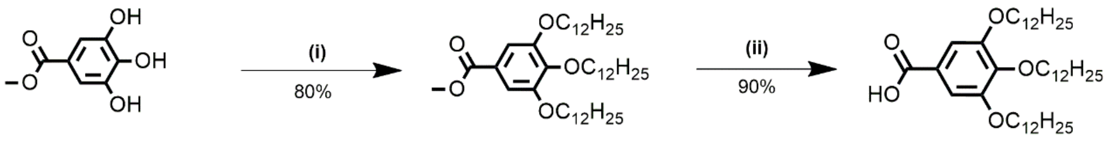

Scheme S2. The synthetic route of **3,4,5-Tris(dodecyloxy)benzoic acid(3,4,5-TDBA)** (i) $C_{12}H_{25}Br$, K_2CO_3, DMF, 80 °C, 16 hr; (ii) EtOH/THF/H_2O = 24/2/1, 90 °C, 8 hr.

Scheme S1 shows the synthetic route of the SD. The SD was synthesized by connecting two hydrophobic arms (**3,4,5-TDBA**) to a pentaerythritol core *via* Steglich esterification. The **3,4,5-TDBA** was synthesized according to **Scheme S2**. Because the Steglich esterification gives better yield when the reactants have good solubility in the solution, instead of directly adding 2 equiv. of the **3,4,5-TDBA** to a pentaerythritol core, compound 1 was first synthesized from pentaerythritol to increase solubility of the pentaerythritol core. 2 equiv. of **3,4,5-TDBA** was then attached to compound 1 to afford the formation of 2. After the deprotection of 2, the formation of the final products SD was done. ^{1}H NMR (400 MHz, $CDCl_3$) δ 7.23 (s, 4H), 4.47 (s, 4H), 4.02 (t, J = 6.6 Hz, 4H), 3.97 (t, J = 6.5 Hz, 8H), 3.70 (s, 4H), 1.83 – 1.71 (m, 12H), 1.50 – 1.44 (m, 12H), 1.37 – 1.23 (m, 98H), 0.88 (t, J = 7.1 Hz, 18H) ppm. ^{13}C NMR (151 MHz, $CDCl_3$) δ

167.30, 153.09, 143.19, 123.81, 108.38, 77.16, 73.73, 69.40, 63.22, 62.90, 46.17, 32.07, 30.49, 29.80, 29.52, 26.26, 22.84, 14.25 ppm. HRFD-Mass (m/z) calcd. $[M+1]^+$ for $C_{91}H_{164}O_{12}$, 1450.2 Da; found 1450.2 Da.

***General Synthetic Procedures for Compounds 4 and* 3,4,5-TDBA**

Compound 4

Compound 4 was prepared from commercially available compound 3. Compound 3 (methyl 3,4,5-trihydroxybenzoate, 54.3 mmol, 10.0 g, 1 equiv.) and dimethylformamide (DMF, 95.0 mL) was placed in a 250 mL two-necked flask. Then 1-bromododecane (222.6 mmol, 53.36 mL, 4 equiv.) and potassium carbonate (K_2CO_3, 244.4 mmol, 33.7 g, 4.5 equiv.) were added to the flask under $N_{2(g)}$. The reaction mixture was stirred at 85 °C for 16 hr. After cooling to rt., the solution was poured into 800 mL of ice water, and subjected to vacuum filtration after being placed in an ice bath for 30 minutes. The resulting solid was recrystallized using an appropriate amount of hot acetone (50 °C), followed by vacuum filtration of the acetone solution. The solvent was removed using a high vacuum system to obtain white solid Compound 4 (yield: 80 %).The ^{1}H-NMR spectra of Compounds 4 was shown in **Figure S1**. ^{1}H NMR (400 MHz, $CDCl_3$) δ 7.25 (s, 2H), 4.0 (m, 6H), 3.88 (s, 3H), 1.85 – 1.71 (m, 6H), 1.50 – 1.42 (m, 6H), 1.26 (m, 48H), 0.88 (t, J = 6.8Hz, 9H) ppm.

***Compound* 3,4,5-TDBA**

Compound 4 (1 equiv., 34.82 mmol, 24 g), Potassium hydroxide (KOH, 87.08 mmol, 4.88 g, 2.5 equiv.) was dissolved in 400 mL EtOH/THF/H_2O solution (v/v/v = 24:2:1). The reaction mixture was stirred at 90 °C for 8 hr under $N_{2(g)}$. After cooling to rt., the solution was poured into 1000 mL of water and placed it in an ice bath for 30

minutes. Then, 12 M concentrated hydrochloric acid (HCl) was slowly added dropwise to the solution until the solution reached a pH of 1.0. The solution was placed in a 4 °C refrigerator for 12 hours to allow the product to precipitate. The precipitate was separated using vacuum filtration and extracted with Hexane. The organic layer was dried over anhydrous $MgSO_4$ and filtered, the filtrate was finally concentrated under reduced pressure and high vacuum system to give compound **3,4,5-TDBA** as a milky white solid (yield: 90 %). The ^{1}H-NMR spectra of compounds **3,4,5-TDBA** was shown in **Figure S2**. ^{1}H NMR (400 MHz, $CDCl_3$) δ 7.29 (s, 2H), 4.00 (m, 6H), 1.85 – 1.78 (m, 6H), 1.46 (m, 6H), 1.26 (s, 49H), 0.90 – 0.85 (t, J = 6.4 Hz, 9H) ppm.

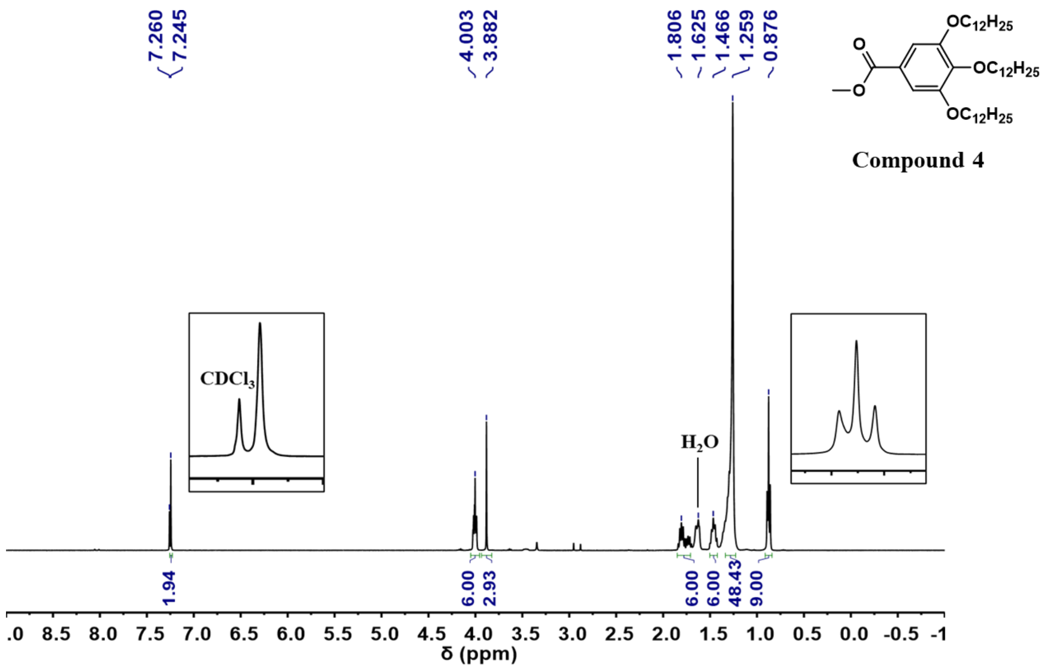

Figure S1. ^{1}H NMR spectra of compound 4 (400 MHz in $CDCl_3$).

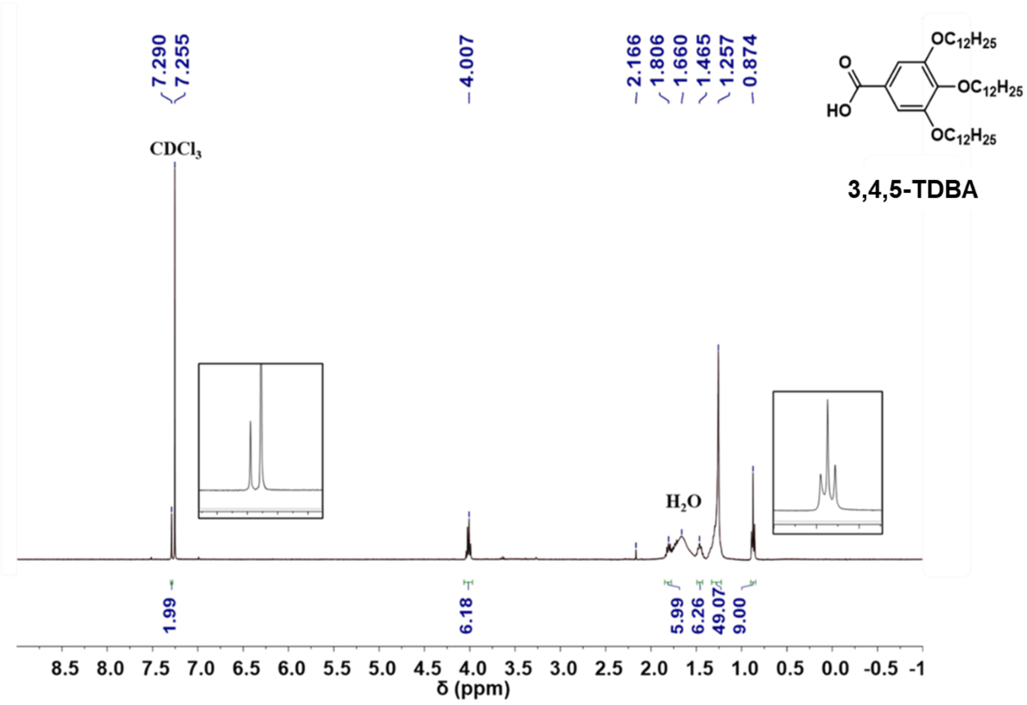

Figure S2. ^{1}H NMR spectra of HRM (400 MHz in $CDCl_3$).

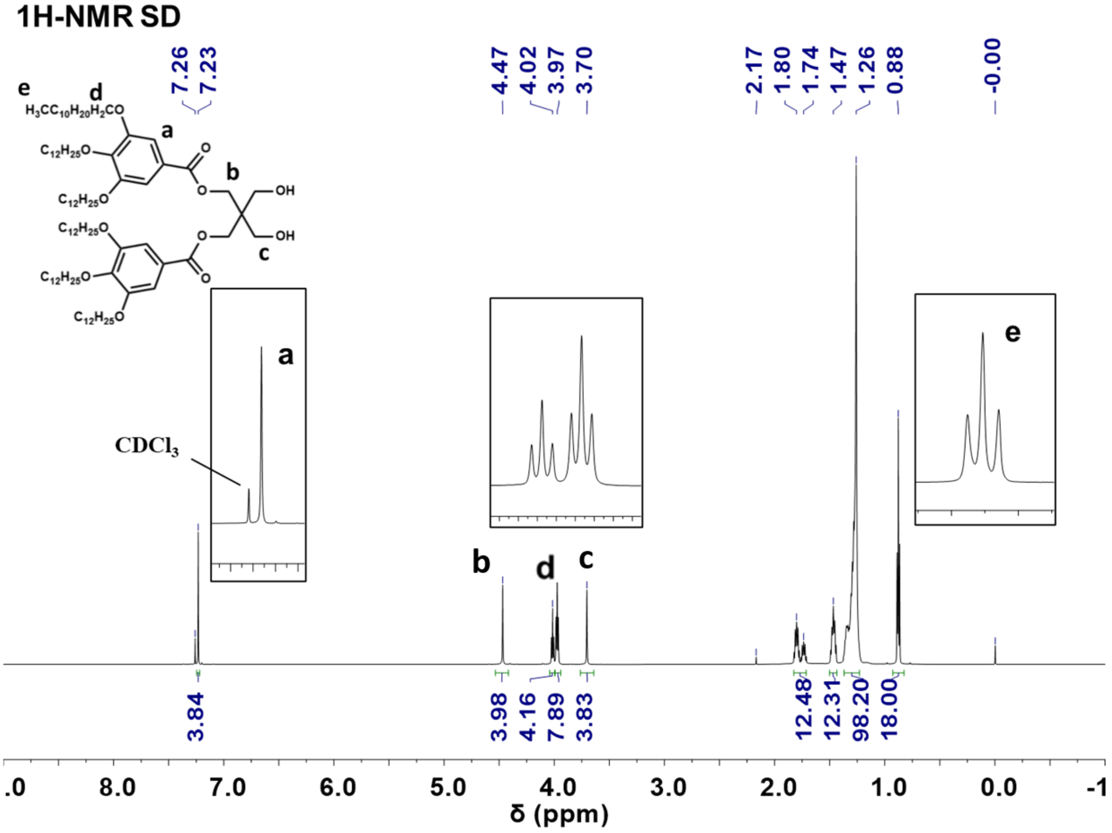

Figure S3. ^{1}H NMR spectra of the SD (600 MHz in $CDCl_3$).

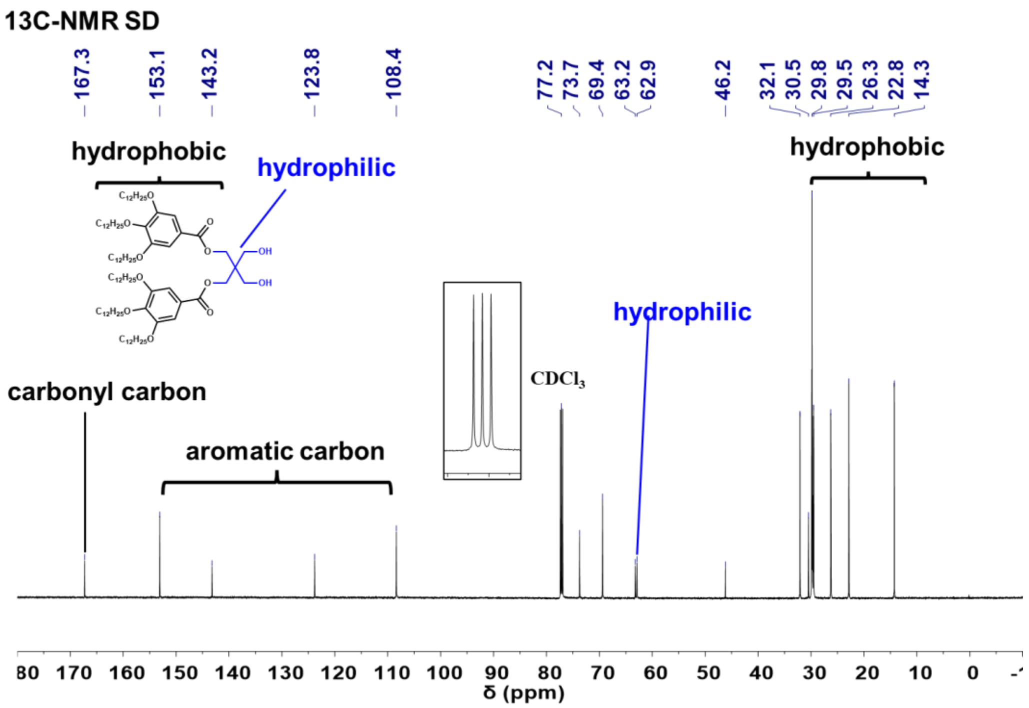

Figure S4. ^{13}C NMR spectra of the SD (151 MHz in $CDCl_3$).

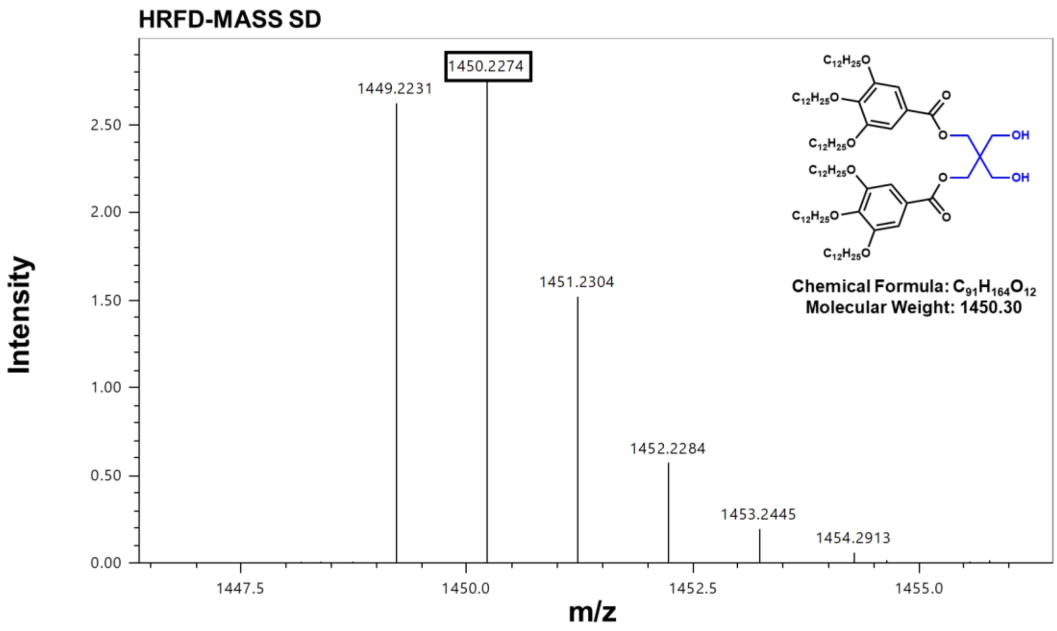

Figure S5. HRFD-Mass spectra of the SD.

2. Structural Characterization of the SD

Cell parameters of the DDQC phase:

$$\boldsymbol{a} = \boldsymbol{b} = \sqrt{\frac{7+4\sqrt{3}}{3}} \times \boldsymbol{d}_{12100} \qquad \text{(Eq. S1)}$$

$$\boldsymbol{c} = 2 \times \boldsymbol{d}_{00002} \qquad \text{(Eq. S2)}$$

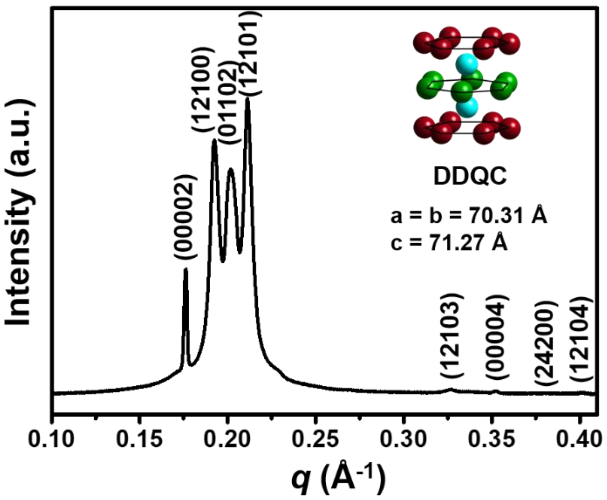

Figure S6. The SAXS pattern of the DDQC phase of the SD with peak assignments and unit cell parameters. The Miller index of the peaks were given according to the literature.[1]

Table S1. List of the q ratio obtained from literature[1] and observed q for σ phase (the dehydrated SD at 50 °C during 1st heating scan) $\boldsymbol{q}$ **ratio** $= \boldsymbol{q}/\boldsymbol{q}_0$, where $\boldsymbol{q}_0$ represents scattering position of the first nonzero diffraction peak.

**Miller indices** $(h_1h_2h_3h_4h_5)$	q_{calc} **ratio**	q_{obs} **(Å^{-1})**	q_{obs} **ratio**
(00002)	1.00	0.1763	1.00
(12100)	1.08	0.1924	1.09
(10102)	1.12	0.2017	1.14
(12101)	1.19	0.2112	1.20
(12103)	1.85	0.3269	1.85
(00004)	2.00	0.3526	2.00
(24200)	2.15	0.3805	2.16
(12104)	2.27	0.4019	2.28

Cell parameters of the σ phase (Tetragonal system):

$$\boldsymbol{a = b = 3\sqrt{2} \times d_{330}} \quad \text{(Eq. S3)}$$

$$\boldsymbol{c = 2 \times d_{002}} \quad \text{(Eq. S4)}$$

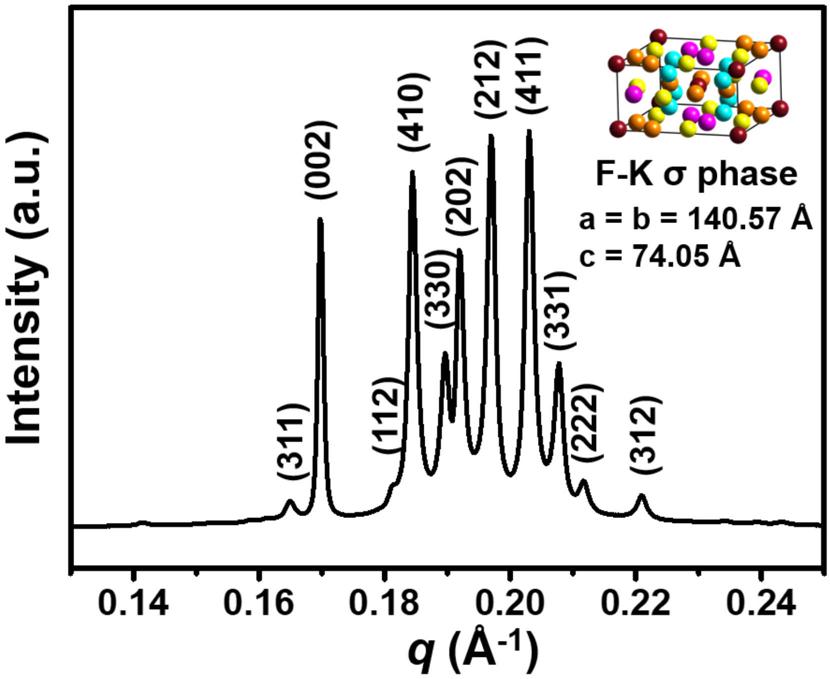

Figure S7. The SAXS pattern of the σ phase of SD with peak assignments and unit cell parameters.

Average number of molecules per polyhedral:

$$M = \frac{V\rho}{30m} \times N_A = \frac{abc\rho N_a}{30m} \quad \text{(Eq. S5)}$$

where V is the size of one σ phase lattice (a = b = 140.57 Å and c = 74.05 Å), ρ is the density of the sample measured at 25 ºC (0.93±0.002 g/cm^3), 30 is the number of spheres in one σ lattice, m is molecular weight of sample (1450.3 g/mol) and N_A is the Avogadro's number (6.022 × 10^{23} mol^{-1}).[2]

Average spherical diameter:

$$d = 2 \times \sqrt[3]{\frac{V}{40\pi}} = \sqrt[3]{\frac{abc}{5\pi}} \quad \text{(Eq. S6)}$$

Calculated q value for σ phase (Tetragonal system):

Miller indices of the tetragonal space group symmetry of $P4_2/mnm$, cell parameters of a = 140.57 Å and c = 74.05 Å, and the equation below are used to calculate q_{hkl} for σ phase.

$$q_{hkl} = 2\pi \times \sqrt{\left(\frac{h^2+k^2}{a^2}\right) + \frac{l^2}{c^2}} \quad \text{(Eq. S7)}$$

Table S2. List of the calculated and observed q for the dehydrated σ phase of SD at 30 °C.

Miller indices (hkl)	q_{calc} (Å^{-1})	q_{calc} ratio	q_{obs} (Å^{-1})	q_{obs} ratio	$\Delta q/q_{calc}$ (%)
(310)	0.1413	1.00	0.1414	1.00	0.04
(221)	0.1523	1.08	0.1525	1.08	0.16
(301)	0.1587	1.12	0.1588	1.12	0.10
(320)	0.1612	1.14	0.1613	1.14	0.11
(311)	0.1649	1.17	0.1650	1.17	0.05
(002)	0.1697	1.20	0.1697	1.20	0.01
(112)	0.1811	1.28	0.1813	1.28	0.09
(410)	0.1843	1.30	0.1844	1.30	0.07
(330)	0.1896	1.34	0.1896	1.34	0.00
(202)	0.1918	1.36	0.1919	1.36	0.05
(212)	0.1969	1.39	0.1969	1.39	0.03
(411)	0.2029	1.44	0.2030	1.44	0.05
(331)	0.2078	1.47	0.2078	1.47	0.00
(222)	0.2116	1.50	0.2116	1.50	0.00
(312)	0.2209	1.56	0.2209	1.56	0.02
(510)	0.2279	1.61	0.2279	1.61	0.00
(322)	0.2340	1.66	0.2343	1.66	0.09
(501)	0.2391	1.69	0.2392	1.69	0.07
(511)	0.2432	1.72	0.2433	1.72	0.04

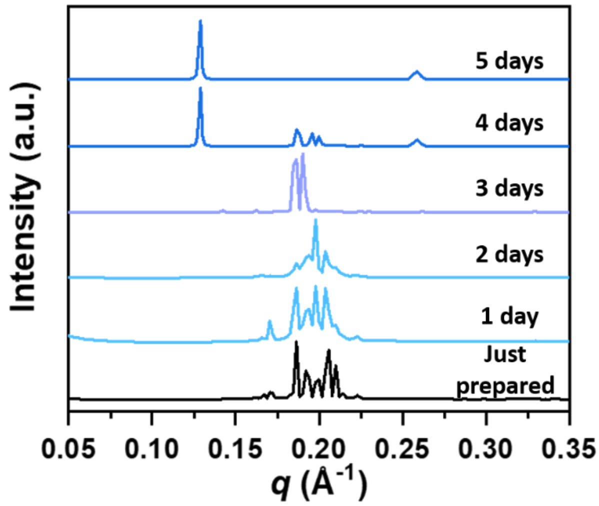

Figure S8. The ex-situ SAXS experiments of the dehydrated σ phase exposed to the 75% RH for 1 – 5 days.

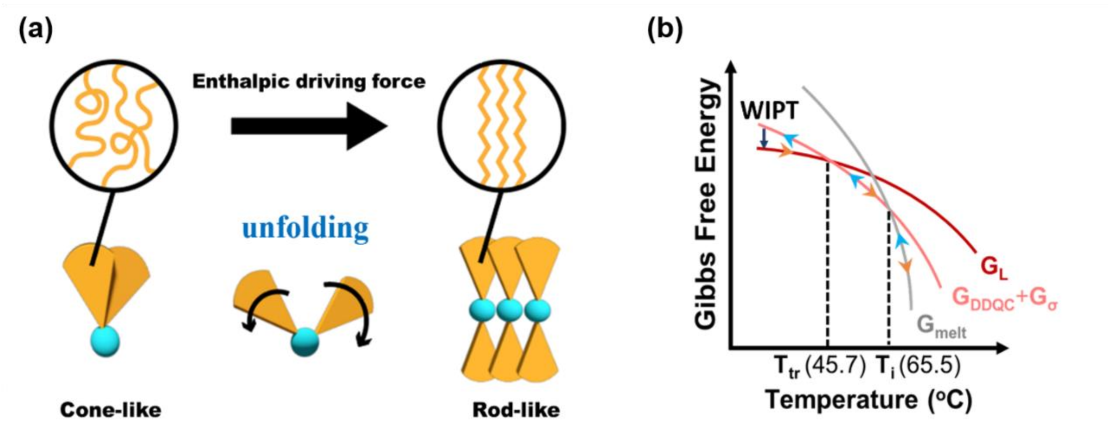

Scheme S3. (a) Illustration the possible unfolding mechanism for the water-induced σ → L phase transition. (b) Illustrative isobaric G–T plot of the phase behavior of SD. The orange and blue arrows show the phase behaviors during the heating and cooling processes, respectively. The black arrow indicates the RT water-induced phase transition, which involves the conformational ordering of the dodecyl chains.

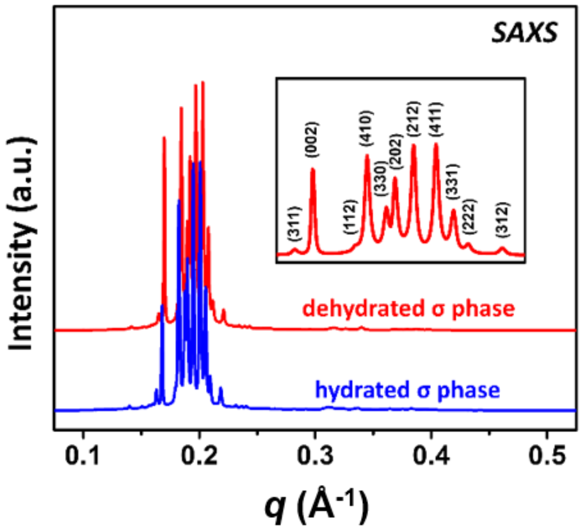

Figure S9. The SAXS patterns of the σ phase resulted from the L phase (the dehydrated σ phase, red trace), and the σ_w phase resulted from the L_w phase (the hydrated σ phase, blue trace).

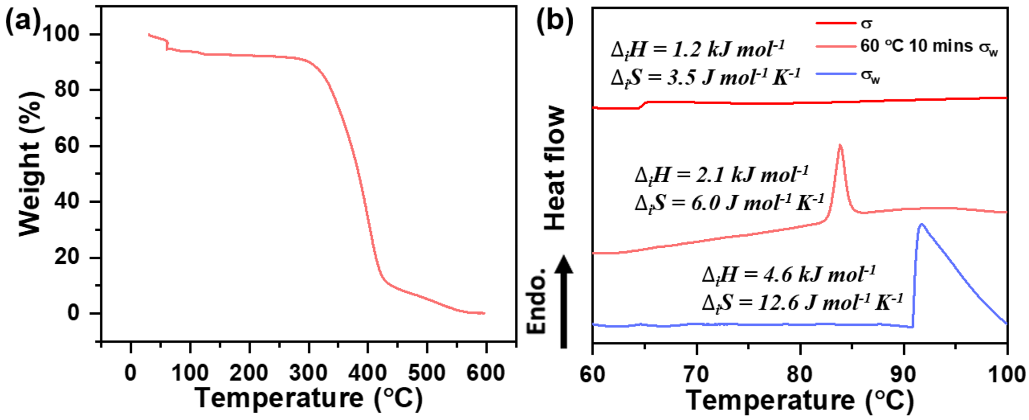

Figure S10. (a) Thermogravimetric analysis result of hydrated SD. (b) DSC thermograms of the σ, σ_w and the σ_w phase that has been thermal annealed at 60 °C for 10 minutes.

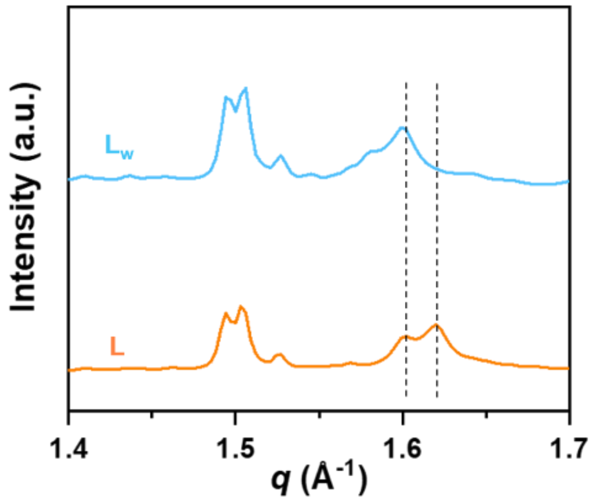

Figure S11. The wide-angle X-ray scattering (WAXS) patterns of the L and L_w phases of the SD.

3. References

(1) Zeng, X.; Ungar, G.; Liu, Y.; Percec, V.; Dulcey, A. E.; Hobbs, J. K. Supramolecular dendritic liquid quasicrystals. *Nature* **2004**, *428*, 157-160.

(2) Feng, X.; Liu, G.; Guo, D.; Lang, K.; Zhang, R.; Huang, J.; Su, Z.; Li, Y.; Huang, M.; Li, T. Transition kinetics of self-assembled supramolecular dodecagonal quasicrystal and Frank–Kasper σ phases in AB n dendron-like giant molecules. *ACS Macro Lett.* **2019**, *8*, 875-881.
